# A novel 3D volumetric method for directly quantifying porosity and pore space morphology in flocculated suspended sediments

**DOI:** 10.1016/j.mex.2022.101975

**Published:** 2022-12-22

**Authors:** TJ Lawrence, SJ Carr, AJ Manning, JAT Wheatland, AJ Bushby, KL Spencer

**Affiliations:** a School of Geography, Queen Mary University of London, London, UK, E1 4NS; b Institute of Science and the Environment, University of Cumbria, Ambleside, UK, LA22 9BB; c School of Biological and Marine Sciences, University of Plymouth, UK, PL4 8AA; d River Restoration Centre, Cranfield University, UK, MK43 0AL; e School of Engineering and Materials Science, Queen Mary University of London, UK, E1 4NS; f School of Earth and Environmental Sciences, Cardiff University, UK, CF10 3AT

**Keywords:** Porosity quantification, Flocculation, Estuary sediments, Pore space

## Abstract

Flocculated suspended sediments (flocs) are found in a variety of environments globally, and their transport and behavior bear substantial importance to several industries including fisheries, aquaculture, and shipping. Additionally, the modelling of their behavior is important for estuarine and coastal flood prediction and defence, and the process of flocculation occurs in other unrelated industries such as paper and chemical production. Floc porosity is conventionally assessed using inferential indirect or proxy data approaches. These methods underestimate floc porosity % by c. 30% and cannot measure the micro-scale complexity of these pore spaces and networks, rendering inputs to models sub-optimal. This study introduces a novel 3D porosity and pore space quantification protocol, that produces directly quantified porosity % and pore space data.

• 3D floc data from micro-CT scanning is segmented volumetrically
• This segmented volume is quantified to extract porosity and several pore space parameters from the floc structure

## Graphical abstract

**Figure:**
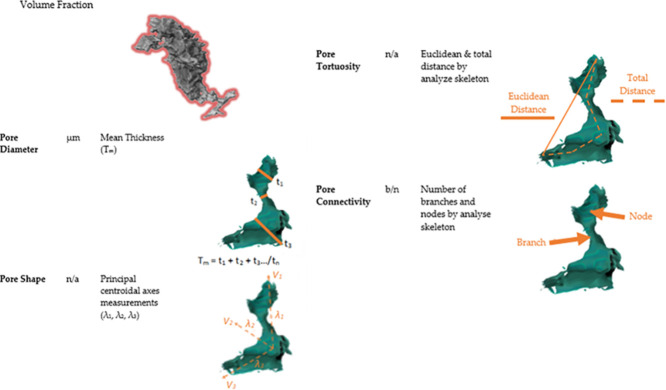

Specifications Table

**Table:** 

Subject area	Environmental Science
More specific subject area	Fluvial Geomorphology
Name of your method	3D Quantification of Porosity and Pore Space in Flocs
Name and reference of original method	N/A
Resource availability	Fiji (ImageJ)

## Method details

### Introduction

Cohesive suspended sediment often exists, and is transported, as flocculated aggregates commonly referred to as “flocs” [1], [2], [3], [4]. Flocs are highly porous, low density, fragile structures that aggregate, disaggregate, and settle in a variety of environments, including estuaries [1,^5^,6]. Flocs contribute to, and facilitate, the exchange and transport of contaminants and pollutants, erosional processes and alternations in fluvial geomorphology in estuarine environments [7], [8], [9], [10], [11].

Porosity occupies a substantial proportion of floc structure, often reported between 70-95% [5,[12], [13], [14], [15]], and is incorporated into the structure during multiple phases of aggregation and disaggregation [16], [17], [18]. Despite the importance and prevalence of porosity in flocs, it is often inferred from effective density values calculated from laboratory settling experiments (using settling columns such as LabSFLOC (^19^) or field data [20], [21], [22], [23], rather than direct observation. This process involves 2D Feret diameter size measurements of a 3D object, assumes structural fractality, and cannot detect individual pore characteristics or pore network complexity [21,[24], [25], [26]]. Consequently, the resultant inferred porosity values are based on a series of inferences and assumptions and miss subtleties in porosity such as pore morphological characteristics. Sediment transport modelling relies on the accuracy of a selection of inputs including, but not limited to, aggregate size and porosity [27], [28], [29], [30]. Without reliable input datasets, model outputs can cause costly mistakes to be made such as the maintenance dredging material mis-location issue at the Port of Rotterdam [31].

3D microtomography, and associated image processing and analysis, are tools originally developed for use in medical and engineering applications [32], [33], [34], [35], but have increasingly been applied to studying sediments [36], [37], [38]. As the process is non-destructive and can accommodate appropriately prepared fragile structures such as flocs, it is a useful method for gaining directly-observed, 3D data of objects that would otherwise prove difficult to examine [36,39]. Image post-processing and analysis tools such as ImageJ/Fiji ^40^ have increasingly been used to investigate biological and geoscientific microtomographic datasets [41], [42], [43].

#### Development of the protocol

ImageJ/Fiji ^40^ is widely used as a package for analysing image data, as it includes a set of tools and plugins that allows 3D volumetric quantification and analysis, including MorphoLibJ [44] and BoneJ [45]. Until recently however, an approach utilizing these tools has not been developed to quantify and analyse flocculated sediments in 3D [46]. We have developed a protocol to post-process, segment, quantify and analyse 3D micro-computed tomography scan data of flocs. This protocol allows a direct 3D volumetric measurement of porosity, which is not possible by any other method.

The protocol includes 5 main stages of processing, that can be adapted to fit alternative object types. Stage one is image stack optimisation, designed to produce a sample dataset with as little noise as possible without losing signal. Stage two involves segmentation into solid floc components, which identifies flocs within the greater sample volume and labels them for sub-sampling. Stage three is the sub-sampling process, where individual flocs are 3D-cropped from the total volume. Stage four is individual floc segmentation, where each cropped floc volume is segmented into solid material, pore space belonging to the floc (divided into effective pore space which is hydraulically connected to the outside of the floc, and isolated pore space that is hydraulically isolated), and external empty/irrelevant space. The final stage is quantification of bulk porosity and individual pore space and pore network characteristics: pore diameter, pore shape, pore tortuosity, and pore connectivity.

#### Applications of the method

This protocol has been developed, and tested, for use in flocs [46], but could be applied to any sediment or aggregate application where structural boundaries are not clear, or where existing methods use assumptions/proxies rather than direct measurements. This is because the protocol presented here directly measures the object in question, rather than sampling the material of an object. This protocol measures the porosity that belongs to a structure, rather than assessing how much void space is present in a material sample. The issue of unclear structural (floc & pore) boundaries is resolved in this protocol by use of a closing ball filter, whereby a rolling maximum fit sphere is applied to binary image data, and “closes off” the physical boundary of the floc suspended in 3D space. This filtering method provides a flexible tool that looks at where pores and pore throats go beyond thresholds enabling hydraulic flow [47], that can be applied to any porous aggregate. As an advantageous method for use instead of proxy or indirect methods, this protocol enables the direct 3D measurement of flocs, but any 3D tomographic data can be used as input, so this method has far wider application potential than flocs alone.

#### Comparison with other methods

CT data-based techniques are reported in studies on soil aggregates [48], [49], [50], where porosity and pore diameter distribution are measured. These techniques typically involve sub-sampling of soils resulting in a cube- or cuboid-bound sample for analysis. This protocol quantifies bulk porosity and pore diameter similarly, however the initial segmentation of porosity volume within the overall volume is performed differently, as flocs do not possess an artificial external boundary to the sample.

**Fig. 1:**
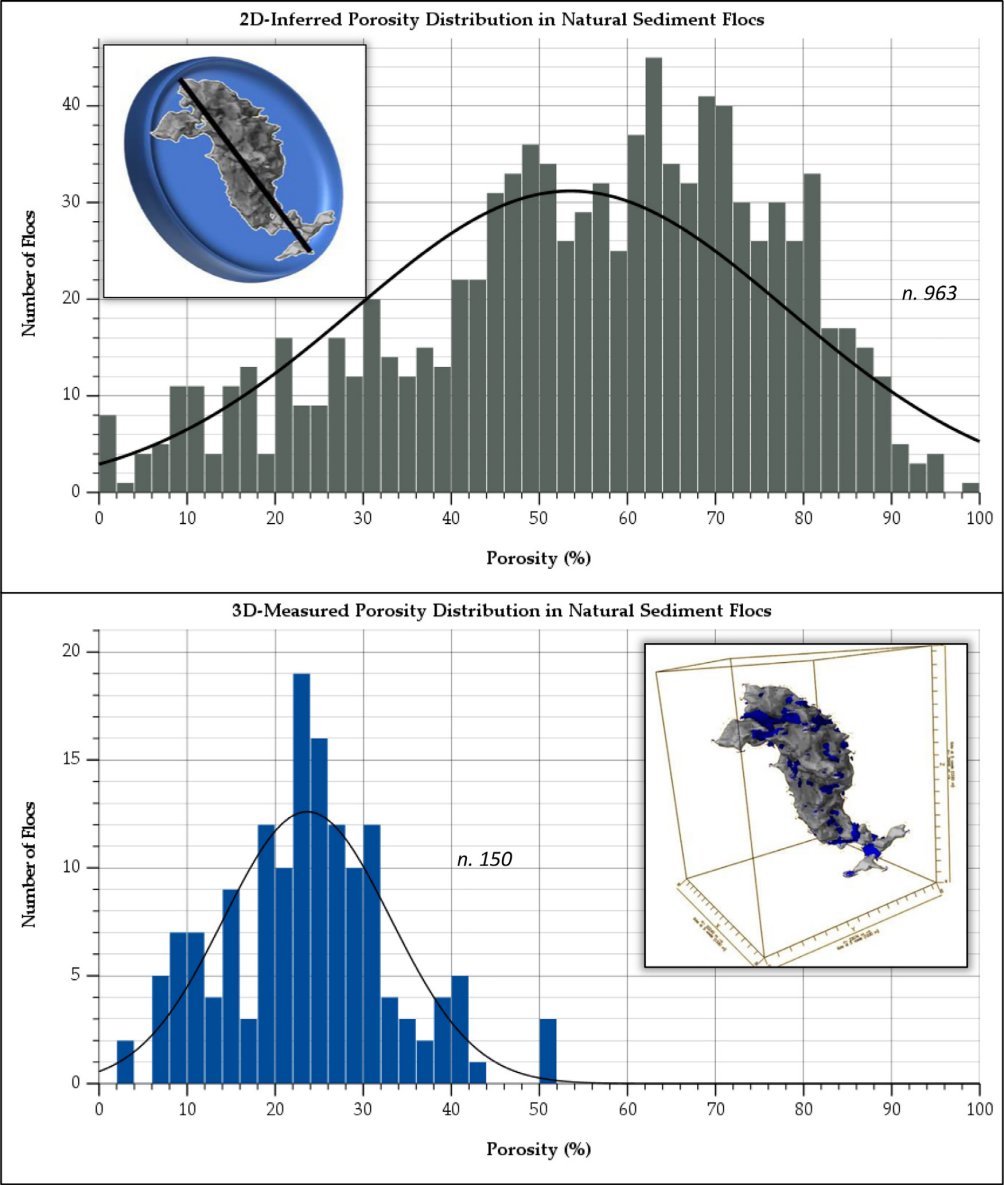
Porosity (%) distribution measured by indirect 2D (top) and direct 3D (bottom) methods. A normal distribution line has been included in both plots. Inset top panel: representation of conventional 2D method applied to a floc. Inset bottom panel: rendered image of 3D-measurement method applied to a natural floc (Lawrence et al. 2022)

The closest analogue in estuarine sediment studies to the data produced by this protocol is the derivation of porosity in flocs inferred indirectly from effective density data gained from settling experiments. A comparison of data obtained using this protocol and the conventional 2D inference method is presented in Fig. 1.

There is a substantial difference in the distributions, median and mode values in the two porosity measurement datasets in Fig. 1. The conventional 2D method data ranged from 0.4-98% with a mean value of 55% and a median of 58%. The 3D data ranged between 4-52%, with mean and median values of 24%. The conventional method uses 2D floc size data, produced from a Feret diameter ellipsoid. This means that much of the space assigned as empty volume belonging to the floc is irrelevant external volume, reducing the effective density value and consequently raising the porosity value.

An advantage of the new protocol presented here is the elimination of this irrelevant external space, meaning that all porosity is directly measured from the floc structure. Additionally, the ability to individually segment pore spaces means that characteristics such as 3D diameter, shape, tortuosity, and connectivity can now be measured, which was previously simply not possible.

#### Experimental design

##### Initial flocculated material sampling

To obtain flocculated sediment samples for μCT preparation, a plankton chamber was placed within the LabSFLOC [19] system to collect material once sediment settling had completed. This flocculated material was then immobilized using agarose gel and kept cool (at 4 degrees Celsius) prior to μCT preparation to minimize bacterial activity and structural alteration.

##### Sample staining during preparation and embedding for μCT

The floc samples used as input data for this protocol were prepared according to the staining and embedding methodology outlined by Wheatland et al. [51].This methodology details the use of glutaraldehyde and formaldehyde fixatives, ethanol and acetone-based dehydration and embedding in Durcupan resin to stabilize the floc samples prior to μCT scanning.

This methodology also involved the use of heavy metal stains (e.g. Uranyl Acetate) to enhance contrast between organic and inorganic constituents of flocs in the μCT scan data. Uranyl Acetate and Osmium Tetroxide were added to the samples during the embedding process, as they offer very good contrast enhancement staining to the bacteria and organic detritus commonly found in estuarine flocs [51], however the suitability of these stains may vary when the sample material differs. Whilst this staining and embedding procedure is not part of the protocol, it affects the raw data required for implementation.

##### Considerations for μCT image acquisition

Floc samples were scanned by μCT using a Nikon Metrology XT-H 225, fitted with a transmission target with a potential focal spot size of 1μm. The scans were performed at a voltage of 150kV and a current of 160μA. These conditions provided the best representation of the floc objects in the resultant image data. Lower voltages are preferable as they facilitate the best sample material discrimination, however a relatively high voltage was required to penetrate the denser materials present within these samples [52]. As a result of using higher voltages the overall wattage increased, causing a change in scan resolution [53] from 1*μ*m to 2.78*μ*m by increasing the noise comparative to signal [54]. A 1mm copper filter was fitted to the *μ*CT during these scans. Filters are included in *μ*CT to absorb low energy X-rays [55] which is important as this makes the X-ray beam more monochromatic. This decreases the impact of beam hardening and ring artefacts in the resultant reconstruction [36,56]. Using X-ray beams that are more monochromatic reduces these issues by reducing the ‘cupping effect’, an issue created in the outer regions of the sample by weaker X-rays being more likely absorbed than higher energy X-rays [36]. The sample scans produced approximately 1600 individual X-ray projections, reconstructed using CTPro3D [57] to produce 16-bit grayscale (65, 536 levels) data volumes for data processing and analysis.

##### Choice of software for data processing

ImageJ & Fiji [40], versions 1.52p and 1.50e, including plugins MorphoLibJ [44] and BoneJ [45] were used to process the data. ImageJ and Fiji are cited for use on sample datasets similar to those in this project [58,59]. BoneJ is a plugin within ImageJ that was designed for use on bone structure in biological sciences [60], but the principles and methods have been applied to soils [43,^61^,62] and by extension can be applied to porous sediments. MorphoLibJ is a suite of mathematically derived morphology methods, designed specifically for use in ImageJ, and has been previously applied to soil structures [63]. For 3D data presentation, Drishti [64] was used, but other visualisation tools are available that may be better suited to different research presentation needs or sample types. Since the protocol was developed, advancements in software (BoneJ v2 [65]) have meant that multithread processing is now possible, so certain processes require less time to complete. A consideration here for protocol users is whether to use the original software that the protocol was designed for, or to use the newer version to save time.

##### Floc sampling within μCT scan data

Sampling choices concerning individual flocs from the output scan data depend on the research question at hand. A list of all flocs in any given scan volume is available to the user (labelled stack from stage 10 of the workflow), and can be filtered accordingly to extract a specific list of flocs that the user is interested in. For example, part of the project that these flocs were analysed within, was concerned with quantifying and visualizing the differences between micro-flocs and macro-flocs as defined by their 2D Feret diameter. Therefore, flocs were sampled in groups surrounding the 25^th^, 50^th^ and 75^th^ percentile of each of the micro-floc (<microns Feret diameter) and macro-floc (> microns Feret diameter) data sub-groups.

## Processing workflow

**Fig. 2:**
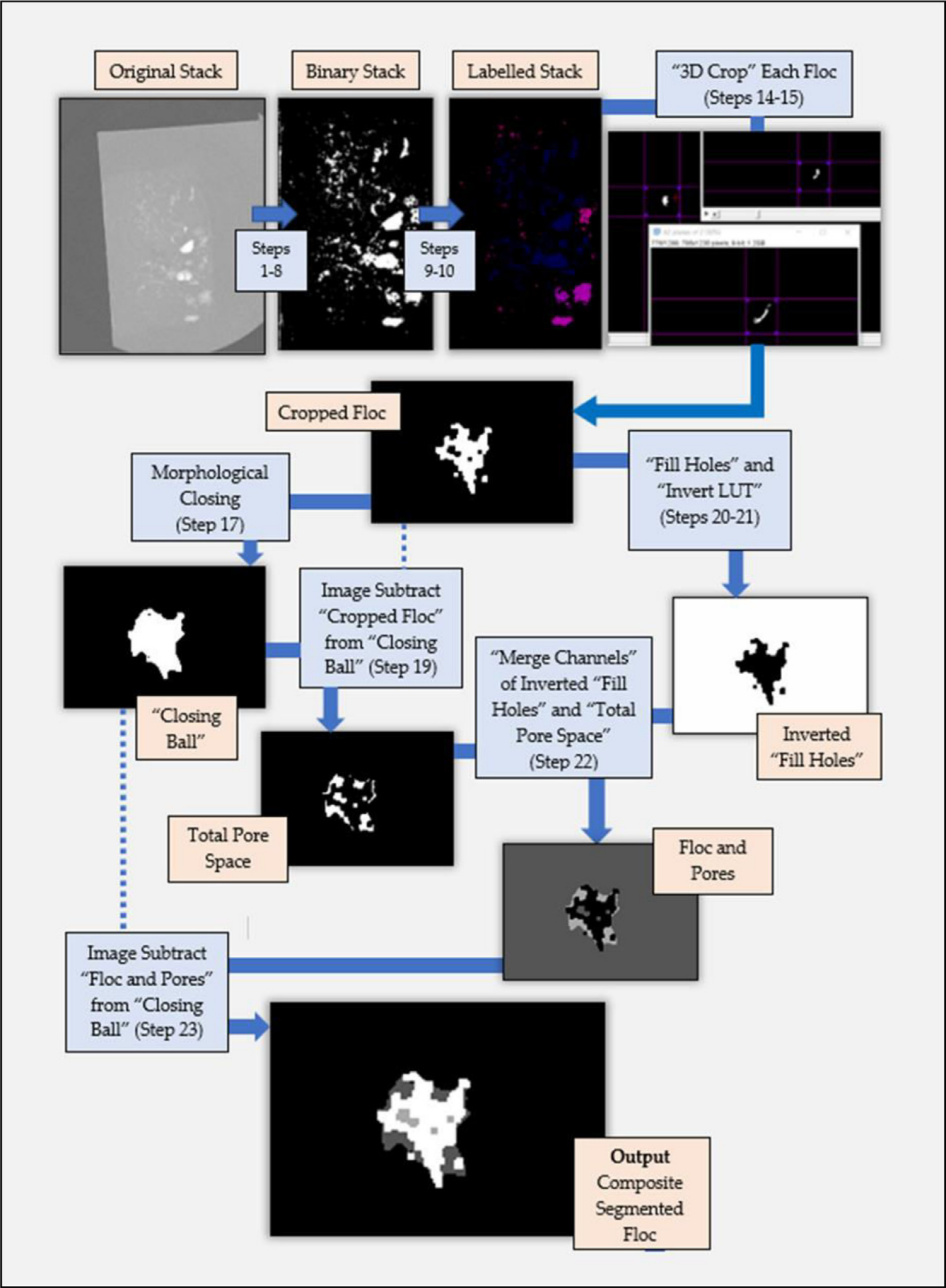
Workflow for data processing from raw data to segmented individual floc sample. Corresponding steps in the procedure are labelled as appropriate.

The data processing and segmentation procedure is summarized in Fig. 2.

### Measurement parameters

**Fig. 3:**
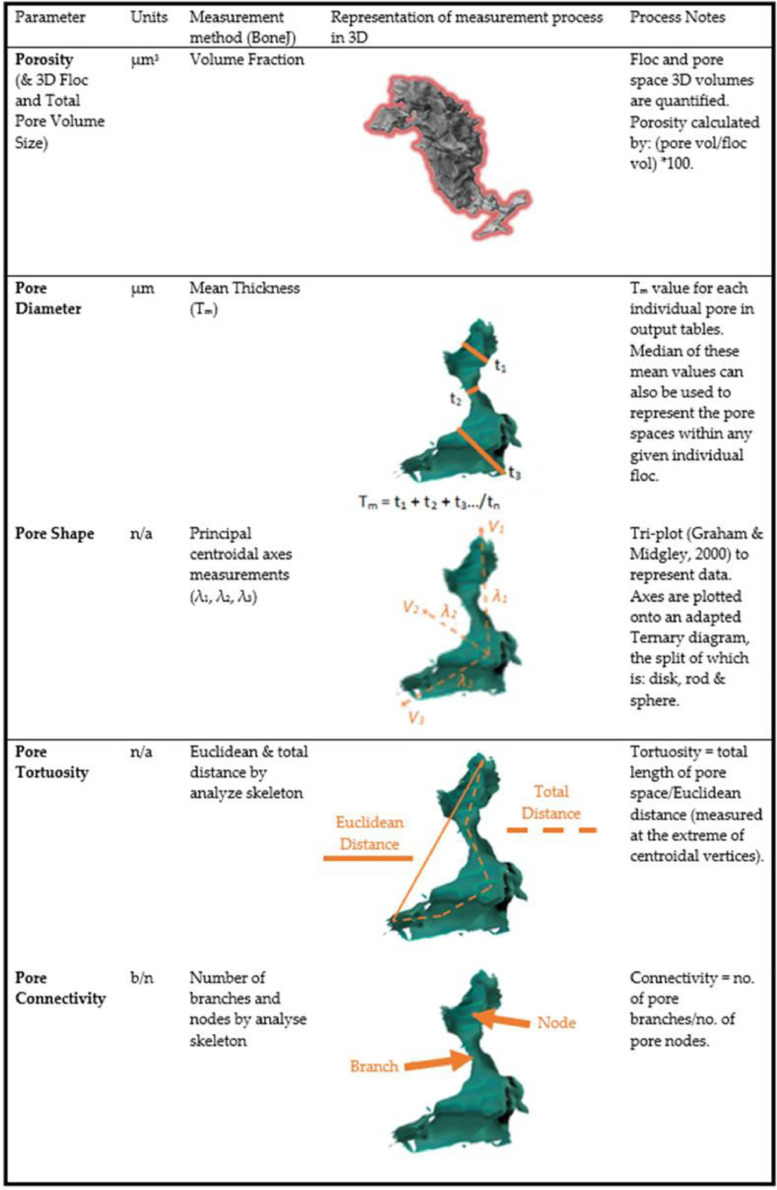
3D pore space features that have been measured, and the method within BoneJ (Doube et al., 2010) by which the data was collected, along with a brief description of how the data was processed into the pore space parameters required.

Bulk porosity measurements were extracted from the dataset using BoneJ's Volume Fraction tool, with the results combined using MS Excel. The individual pore space characteristics were quantified by using several features in ImageJ applied to the *effective* and *isolated pore space* volumes. The pore diameter data was provided by the ‘thickness’ output from the ‘analyse particles’ process. Pore space tortuosity and connectivity measurements were enabled by using ImageJ's ‘skeletonize’ and ‘analyze skeleton’. Tortuosity was calculated by dividing the total branch running distance by the Euclidean distance for each pore. The pore connectivity was calculated by dividing the number of nodes by the number of branches in the pore network skeleton structure. The pore space shape assessment used centroid measurements gathered from the use of analyse particles as an input to an adapted version of Tri-Plot [66]. These processes are summarized in Fig. 3.

#### Limitations

This protocol is designed to extract porosity data on the micro-scale (>5μm), and as such will miss nano-porosity present in the aggregate structure. However, hydrodynamically, water cannot move through spaces smaller than the micro-scale due to hydrostatic forces, so this would not be an issue if the research focus was purely hydrodynamic. This is simultaneously a strength of this work, as we are defining and quantifying hydrodynamically effective porosity here, which affects floc functionality. Work has previously been published considering nano-scale voids in flocculated structures [38], and although these pores have the potential to make up a substantial proportion of the porosity in a floc, natural EPS can be seen to fill the nm- and μm-scale pore spaces in the floc matrix, thus not all nano-porosity is viable.

To overcome the issue of nano-porosity being missed by the μCT method, a technique suited to nano-scale analysis would need to be employed such as FIB-SEM. A correlative approach has been published [38] that can bridge the gap between the micro-scale and the nano-scale. This means that the protocol can be adapted to quantify and analyse porosity across the complete nm- to mm-scale range.

## Materials

### Data

• Reconstructed micro-computed tomography data.

### Equipment

• Computer capable of processing large image datasets, with ImageJ/Fiji installed with the relevant plugins (MorphoLibJ, BoneJ).

### Procedure

+Critical+ ImageJ Macro Recorder can be used to track, record, and save procedures as macros and scripts, that can then be used to batch process (https://imagej.net/scripting/batch) numerous image stacks in organized folders.

Launching ImageJ and opening data (Timing 1 min)

1. Launch the ImageJ (v1.52p) application.
2. Open image stack data (TIFF format). This is done by selecting the *File > Import > Image Sequence* command path. ?Troubleshooting?

Image stack optimisation (Timing 5 mins)

3. Adjust brightness/contrast to best view floc material. This is done by selecting the *Image > Adjust > Brightness/Contrast* command path. For the resin type and μCT scan settings used in our experiments, the values in the resultant window were set to min: 6000, max: 10,000 GL (grayscale level). These values eliminate high attenuating artefacts in the image data (>10,000 GL), and resin block material (∼5000-6000 GL), while retaining all floc material.
4. Apply a median 3D filter. To complete this action, select the *Process > Filters > Median 3D* command path. A radius of 2 × 2 × 2 pixels was selected for our sample.
5. Threshold the image stack. This is done by selecting the *Image > Adjust > Threshold* command path, and in the resultant window tick the “stack histogram” check box before setting the threshold value to 16,000 GL.
6. Crop the dataset to remove empty space surrounding the relevant floc material, reducing file size and processing load. This is done by selecting the *Image > Transform > Rotate* command path. In the popup window, select the “preview” checkbox and use the slider to rotate the sample block into an orientation suitable for encapsulation using a rectangle. Apply a rectangle by dragging the cursor across the image that encapsulates all sample material and then use *Image > Crop*.
7. Binarize the dataset. This is done by selecting the *Process > Binary > Make Binary* command path.
8. Erode and dilate the dataset. Select *Process > Binary > Erode*, and, using the resultant image stack, select *Process > Binary > Dilate*. This process further reduces the noise in the dataset. Save this image stack.

Labelling floc samples within the image stack (Timing 1 min)

+Critical+ This and the two subsequent sections use ImageJ v1.50e (2015)

9. Using the now binarized image stack, set the scale by selecting the *Analyze > Set Scale* command path. This scale will be dependent on the μCT scan resolution that produced the raw data. ?Troubleshooting?
10. Run particle analysis and labelling on the image stack. This is done by selecting the *Plugins > BoneJ > Analyze > Particle Analyser* command path. In the popup menu, tick “enclosed volume”, set minimum voxels to a reasonable size to screen tiny particles and noise from the dataset (we used 250 voxels/equivalent in μm), tick “show particle stack”, leave everything else unticked. Retain the results window for step 13.

Isolating individual flocs within the population and identifying sub-sampling targets (Timing 30 mins)

11. Using the labelled image stack from step 10, convert to 8-bit by selecting *Image > Type > 8-bit*, and run simple segmentation by selecting *Plugins > 3D > 3D Simple Segmentation*.
12. Set the scale by selecting *Analyze > Set Scale* and then open 3D manager by selecting *Plugins > 3D > 3D Manager.* Click “add image” and this will load each labelled floc into the 3D manager as a separate ‘image’. In the 3D manager options menu, opened by clicking *Plugins > 3D > 3D Manager Options*, tick the boxes “Feret (unit)”, “Volume (unit)”, and “Centroid (unit)”, and click “Measure (3D)”.
13. Using spreadsheet software (such as MS Excel), combine the results data from the Particle Analyser and 3D manager, and order by the volume column. Each individual floc has an ID number, and the individual floc data from each results source can be matched by centroid values. Use this list to select flocs for sub-sampling.

Cropping individual flocs (Timing 2 mins per floc)

14. For each floc to be cropped, reopen the labelled floc stack from step 10. Scroll to the end of the stack, open thresholding by selecting *Image > Adjust > Threshold*, click “set” and enter the floc ID number in both value boxes, then click “apply”. This will produce an image stack that contains only the floc that has been selected. ?Troubleshooting?
15. Use the Crop 3D plugin (*Plugins > Stacks > Crop 3D)* to narrow the selection to contain only the floc material (plus a small surrounding empty region) by using each of the x, y and z plane crop tools. Save the image stack in an appropriate folder, into which further stacks of the same floc can be saved during further stages of processing.

Segmenting porosity in individual flocs (Timing 8 mins per floc)

Note: This section can be automated using ImageJ macros to segment several flocs without user interference

+Critical+ The remainder of the protocol is carried out using ImageJ v1.52

16. Open the binary volume of the floc in ImageJ, by selecting *File > Import > Image Sequence*.
17. Select *Plugins > MorphoLibJ > Morphological Filters (3D)*, click “closing” as operation, “ball” as element shape, and define x/y/z radius that will determine ball size (4/4/4 equated to a ∼10 μm closing ball in our data). Save the output in an appropriate folder.
18. Open another window containing the original cropped floc from step 16 (*File > Import > Image Sequence*) and select *Process > Image Calculator*.
19. In the dialogue box, ‘Image1’ is the result of step 17, ‘Image2’ is the reloaded binary stack from step 18, the operation is “subtract”. This produces the total pore space of the floc. Save the resulting image stack in an appropriate folder.
20. Open the cropped floc by repeating stage 16, then select *Process > Binary > Fill Holes*. Save this output in an appropriate folder and then reopen it in ImageJ (*File > Import > Image Sequence*).
21. Invert the LUT of the output window by selecting *Image > Lookup Tables > Invert LUT*. Save this output as an inverted fill holes stack in an appropriate folder. Open the total pore network stack from stage 19 (*File > Import > Image Sequence*).
22. Select *Image > Colour > Merge Channels*, using the drop-down menus, set “C1” as the total pore space stack from stage 19 and “C2” as the inverted fill holes output from stage 21. Untick “create composite”, tick “keep source images” and “ignore source LUTs”. Select resultant window and click *Image > Type > 8-bit.*
23. Open the outputs of stages 17 and 22 (*File > Import > Image Sequence*), and then select *Process > Image Calculator*. In the dialogue window, “Image1” is the stage 17 output and “Image2” is the stage 22 output, the operation is “subtract”. Click “OK” and the resulting output window contains a segmented single floc containing 4 phases: solid floc material, total pore space, effective pore space and isolated pore space. Save this output.

**Fig. 4:**
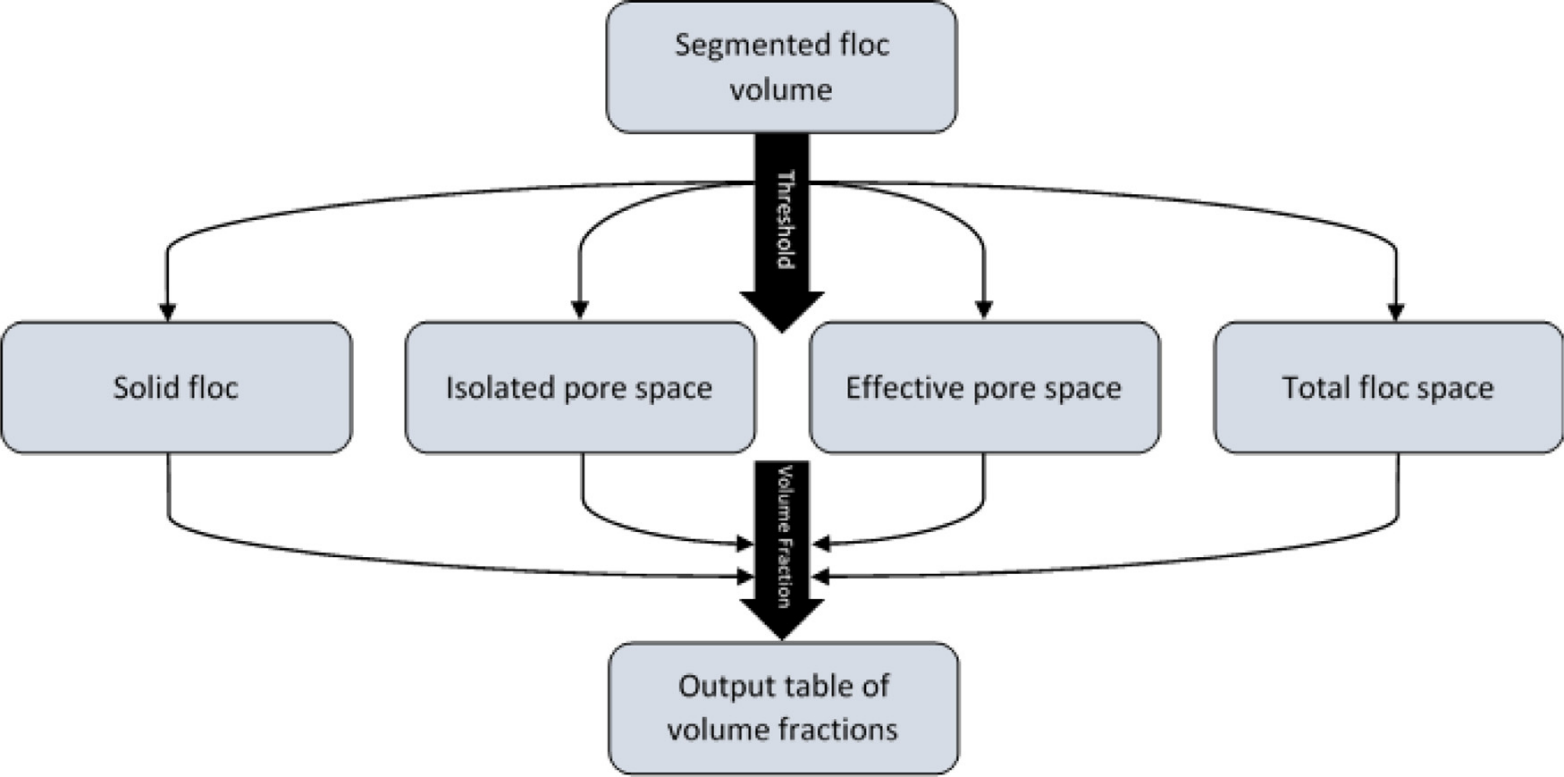
Flow diagram outlining the steps taken during the BoneJ volume fraction analysis of bulk porosity values.

Quantification of bulk porosity (Timing 10 mins)

24. Open the outputs of stages 17 and 23 (*File > Import > Image Sequence*). Repeatedly, select *Plugins > BoneJ > Fraction > Volume Fraction* to calculate the number of μm[3] for each of the following volumes: overall floc space from the stage 17 output, and isolated pores, effective pores, and solid floc from the stage 23 output.
25. The resulting output table content can then be transferred into a spreadsheet to facilitate the calculation of each % as a fraction of the total floc space (e.g. total porosity, effective porosity and isolated porosity).

Quantification of pore space morphology (Timing 5 mins per volume to be quantified (i.e. 5 flocs with all pore volumes quantified will take 75 mins))

26. Perform these steps for each segmented volume (total pores, effective pores, and isolated pores) in each individual floc, to produce a full dataset. Open the image stack (*File > Import > Image Sequence*).
27. Select *Plugins > BoneJ > Analyze > Particle Analyser*, and in the dialogue window, tick the boxes for “Thickness”, “Ellipsoids”, and “Enclosed Volume”, and ensure all other boxes are not ticked. The results of this process provide the data for pore diameter (thickness), pore volume (enclosed volume) and pore shape (ellipsoid radii). Save the results window to open later in an appropriate statistics software.
28. Open the volume again (*File > Import > Image Sequence*). Select *Plugins > Skeleton > Skeletonize (2D/3D)*, which produces a skeletonized version of the pore network within the image volume. Save this image stack in an appropriate folder.
29. Using the skeletonized stack from stage 28, find tortuosity and connectivity by selecting *Analyze > Skeleton > Analyze Skeleton (2D/3D)*. In the dialogue box select “lowest intensity voxel” as the ‘Prune cycle method’ and tick the boxes for “prune ends” and “show detailed info”, ensuring other boxes are not ticked. The resulting output table provides the Euclidean and total running distances to enable calculation of pore tortuosity. The summary output table provides the number of junctions/nodes and number of branches to calculate connectivity. Save these results windows to open later in an appropriate statistics software.

## Troubleshooting

Several stages of the protocol procedure can experience errors that need to be rectified to produce a successful outcome. The solutions to these issues are presented below (Table 1).

**Table 1:** Troubleshooting table, listing the relevant step of the protocol, problem and solution to common issues that can occur whilst completing the protocol.

Step(s)	Error	Possible Reason	Solution
2	ImageJ cannot import the image file.	A limited selection of image formats is supported by ImageJ (JPEG, TIFF, BMP, PNG, and GIF).	Ensure data to be imported is saved as one of the appropriate formats. Check up to date list at https://imagej.nih.gov/ij/features.html.
9-15	ImageJ options/menus do not appear to contain the correct set of options/processes as listed in the protocol.	An inappropriate software version of ImageJ is open.	Use ImageJ v1.50e to complete steps 9-15, and v1.52p to complete the rest of the protocol.
14	Thresholding range does not include desired data.	ImageJ sometimes does not recognise the range of values in the entire stack unless the user scrolls to the final image slice before thresholding.	Close the thresholding window, scroll to the end of the image stack and repeat stage 14 as appropriate.

### Anticipated results

**Fig. 5:**
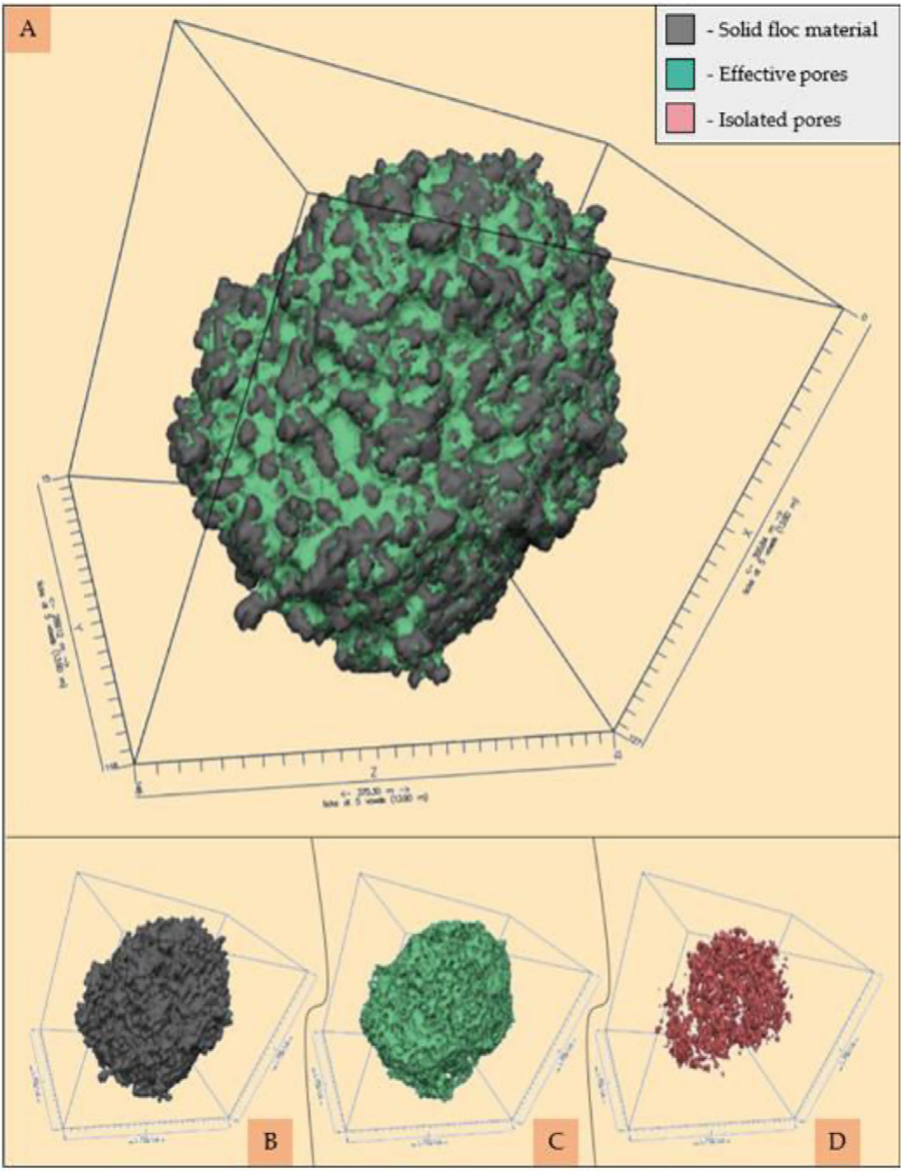
Visual representation using Drishti (Limaye, 2012) of floc constituents by their 3D volume, including the total floc space (A); the solid floc (B); the effective pore volume (C) and the isolated pore volume (D).

The protocol will produce several outputs including segmented 3D floc volumes, and a set of gross porosity % values, pore morphology data and pore network parameter data for each individual floc. The segmented 3D floc volume will comprise a selection/combination of solid floc volume, total pore space volume, effective pore space volume and isolated pore space volume (Fig. 5). Figure 5 should be placed here, prior to the statement regarding further analysis (below)

The raw output data need to be analysed in the following ways to obtain meaningful results:

#### Gross porosity %

The BoneJ results tables cannot be saved as a results window in ImageJ because they are java-based, so the data must be copied out into alternative software (e.g. MS Excel). Once this has occurred, a simple equation can be applied to gain porosity % data: (total pore volume/total floc volume) * 100.

#### Pore size (diameter and volume)

All other pore and pore network analyses can be performed in any stats software/package (SPSS, MS Excel, R). The analyze particles output from BoneJ contains columns titled “thickness”, “SD thickness” and “max thickness” and these are the pore diameter measures. The pore volume data is the “enclosed volume” section of the analyze particles output.

#### Pore shape

The ellipsoid radii values from the analyze particles output can be entered into a clastic tri-plot [66] to gain pore shape data.

#### Pore network characteristics (tortuosity and connectivity)

Pore tortuosity can be calculated from the detailed analyze skeleton output by dividing the total pore length by the Euclidean distance. Connectivity is calculated by using the analyze skeleton summary output, dividing the number of branches by the number of nodes (Fig. 4).

## Ethics statements

No statements were relevant to this work

## Funding

This work was supported by the 10.13039/501100019151NERC grants NE/M009726/1 and NE/N011678/1.

## CRediT authorship contribution statement

**TJ Lawrence:** Methodology, Software, Validation, Formal analysis, Investigation, Writing – original draft, Visualization. **SJ Carr:** Methodology, Software, Writing – review & editing. **AJ Manning:** Methodology, Resources, Writing – review & editing. **JAT Wheatland:** Methodology, Investigation. **AJ Bushby:** Conceptualization, Supervision. **KL Spencer:** Conceptualization, Supervision, Writing – review & editing, Project administration, Funding acquisition.

## Declaration of Competing Interest

The authors declare that they have no known competing financial interests or personal relationships that could have appeared to influence the work reported in this paper.

## Data Availability

Data will be made available on request.
